# North Atlantic Deep Water Production during the Last Glacial Maximum

**DOI:** 10.1038/ncomms11765

**Published:** 2016-06-03

**Authors:** Jacob N. W. Howe, Alexander M. Piotrowski, Taryn L. Noble, Stefan Mulitza, Cristiano M. Chiessi, Germain Bayon

**Affiliations:** 1 Department of Earth Sciences, University of Cambridge, Cambridge CB2 3EQ, UK; 2 Institute for Marine and Antarctic Studies (IMAS), University of Tasmania, Hobart, Tasmania 7001, Australia; 3 MARUM-Center for Marine Environmental Sciences, University of Bremen, Leobener Strasse, D-28359 Bremen, Germany; 4 School of Arts, Sciences and Humanities, University of São Paulo, Av. Arlindo Bettio 1000, CEP03828-000 São Paulo SP, Brazil; 5 Institut Français de Recherche pour l'Exploitation de la Mer (IFREMER), Unité de Recherche Géosciences Marines, F-29280 Plouzané, France

## Abstract

Changes in deep ocean ventilation are commonly invoked as the primary cause of lower glacial atmospheric CO_2_. The water mass structure of the glacial deep Atlantic Ocean and the mechanism by which it may have sequestered carbon remain elusive. Here we present neodymium isotope measurements from cores throughout the Atlantic that reveal glacial–interglacial changes in water mass distributions. These results demonstrate the sustained production of North Atlantic Deep Water under glacial conditions, indicating that southern-sourced waters were not as spatially extensive during the Last Glacial Maximum as previously believed. We demonstrate that the depleted glacial δ^13^C values in the deep Atlantic Ocean cannot be explained solely by water mass source changes. A greater amount of respired carbon, therefore, must have been stored in the abyssal Atlantic during the Last Glacial Maximum. We infer that this was achieved by a sluggish deep overturning cell, comprised of well-mixed northern- and southern-sourced waters.

## Abstract (web summary)

The nature of the overturning circulation in the Atlantic Ocean during the Last Glacial Maximum remains a topic of contention. Here, using neodymium isotope measurements, the authors demonstrate that North Atlantic Deep Water was produced under glacial climate conditions.

## Graphical abstract

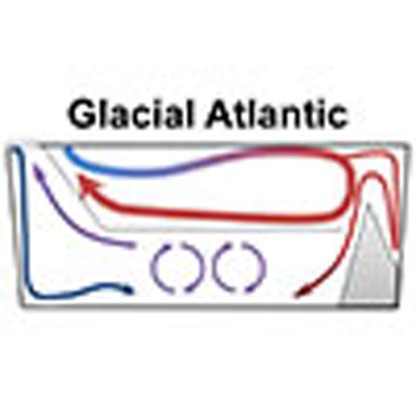

Changes in Atlantic Meridional Overturning Circulation are important in controlling glacial–interglacial climatic shifts due to their role in regulating heat transport in the surface ocean and carbon storage in the deep ocean^1^,^2^. Nutrient-based proxy reconstructions suggest that overturning circulation in the Atlantic during the Last Glacial Maximum (LGM) was different to that of the modern ocean^3^,^4^,^5^. These nutrient proxy reconstructions are often interpreted as indicating that during the LGM North Atlantic Deep Water (NADW) shoaled to form Glacial North Atlantic Intermediate Water (GNAIW) and was completely replaced by southern-sourced water in the deep Atlantic (>2.5 km)^6^,^7^. However, elucidation of the water mass distribution in such reconstructions is complicated by the fact that the nutrient content of deep waters may vary independently of their source and dynamics^8^; meanwhile, modelling studies of glacial Atlantic overturning have produced conflicting results^9^.

In the modern ocean Antarctic Bottom Water (AABW), which is formed in the high latitude Southern Ocean, represents a major inefficiency in the biological pump—the name given to the biologically mediated processes which sequester carbon in the deep ocean. In this region, deep waters are upwelled to the surface, allowing dissolved carbon to be outgassed to the atmosphere, but these waters sink again before their nutrient load can be fully consumed by marine organisms, thus they are said to have a high-preformed nutrient concentration^10^. The formation of NADW and AABW, and their resultant proportions in the deep ocean thus controls the preformed nutrient concentration of the deep ocean, which, in part, controls atmospheric pCO_2_ (ref. 11). Ice core records show that atmospheric CO_2_ concentrations rose by ∼90 p.p.m. between the LGM and the Holocene^12^. However, considered in isolation, the replacement of high-preformed nutrient AABW with low-preformed nutrient NADW, which has been suggested to have occurred across the last deglaciation, would have increased the efficiency of the biological pump and thereby decreased atmospheric CO_2_ (refs 11, 13). If our current understanding of glacial–interglacial circulation changes is correct then other parts of the carbon cycle must have counteracted the effects of switching from the GNAIW to NADW mode of deep water formation across the deglaciation. Alternatively, nutrient proxy reconstructions of the Atlantic may be recording carbon cycle changes rather than differences in water mass sourcing.

Neodymium isotopes are a water mass tracer independent of biological processes, allowing deconvolution of changes in water mass sourcing and nutrient regeneration^14^. Modern NADW has a characteristic *ɛ*_Nd_ value (normalized ^143^Nd/^144^Nd ratio in parts per ten thousand) of −13.5 (ref. 15) while seawater in the deep Southern Ocean shows *ɛ*_Nd_ values around −8.5 (ref. 16), because it has a greater proportion of Pacific Deep Water (PDW) which has an *ɛ*_Nd_ of −3.5 (ref. 17). Away from continental margins water masses throughout the deep ocean reflect the quasi-conservative mixing of these deep water masses^14^. Planktic foraminifera from sea-floor sediment cores have been shown to successfully preserve bottom water *ɛ*_Nd_, thereby offering an archive of past seawater *ɛ*_Nd_ (ref. 18).

Here we present Holocene and LGM seawater *ɛ*_Nd_ reconstructions, based on foraminiferal *ɛ*_Nd_ measurements from 24 cores (Supplementary Table 1, Supplementary Fig. 1) spanning from 46° S to 40° N in the Atlantic Ocean. These reconstructions are used to decipher the nature of changes in Atlantic water mass distributions between glacial and interglacial conditions. Although the LGM reconstruction shows more radiogenic neodymium isotope values than the Holocene profile, the observation of less radiogenic values in the deep North Atlantic than the deep South Atlantic indicates that NADW was produced under glacial conditions. When compared with benthic foraminiferal carbon isotope values, the neodymium isotope measurements reveal that more respired organic carbon was stored in the deep Atlantic Ocean during the LGM than in the Holocene.

## Results

### Core top foraminiferal *ɛ*_Nd_ versus seawater *ɛ*_Nd_

**Figure 1:**
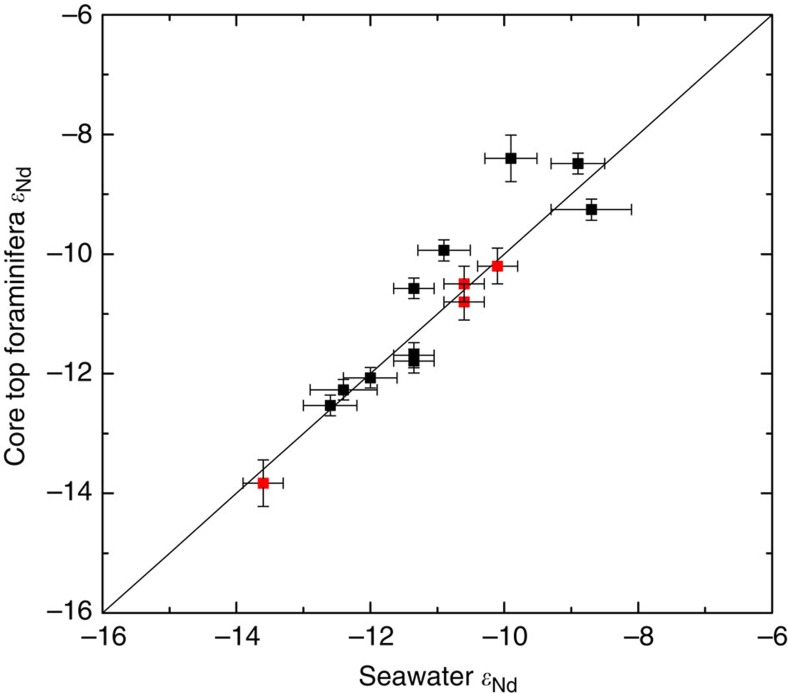
Core top foraminiferal *ɛ*_Nd_ versus seawater *ɛ*_Nd_. Modern open Atlantic seawater^15^,^16^,^55^,^56^ *ɛ*_Nd_ cross plotted against Holocene Atlantic *ɛ*_Nd_ measurements made on uncleaned planktic foraminifera in this work (black squares; Supplementary Table 2) together with previously reported foraminiferal values used in this work (red squares; Supplementary Table 3)^18^,^56^. Error bars are 2σ external errors. A 1:1 line is also plotted.

Figure 1 cross plots the Holocene foraminiferal *ɛ*_Nd_ values (Supplementary Tables 2 and 3) against the nearest available published seawater *ɛ*_Nd_ measurements. Data points were only included in the cross plot if the seawater measurements were within both 10° of latitude and longitude and within 500 m depth of the core site. Most of the data points on the cross plot are within error of the 1:1 line, indicating that foraminifera are faithfully preserving seawater *ɛ*_Nd_. Despite the distance criteria outlined above being applied, the three data points which are outside of error of the 1:1 line are likely due to the seawater data not being of sufficiently close proximity to represent the water mass bathing the core sites. This effect is likely to be particularly prominent in site locations that are near water mass boundaries.

### Holocene Atlantic *ɛ*_Nd_ reconstruction

**Figure 2:**
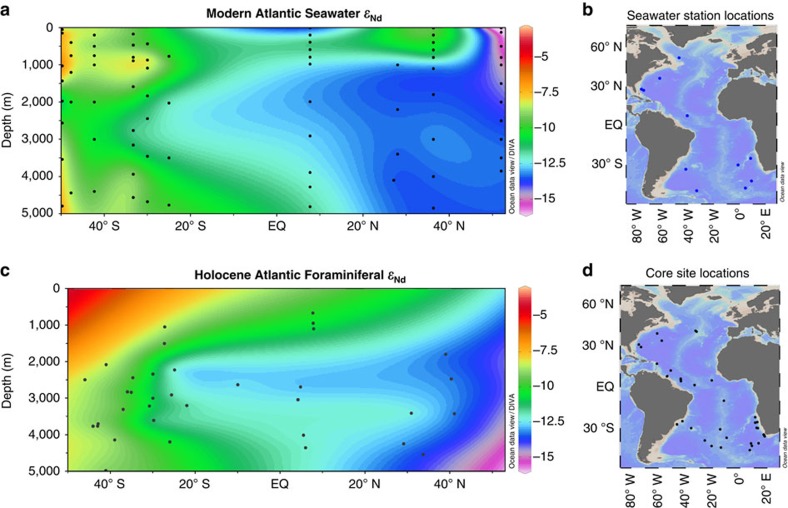
Atlantic seawater *ɛ*_Nd_ and Holocene *ɛ*_Nd_ reconstruction. (**a**) Modern seawater *ɛ*_Nd_ profile from 50° S to 53° N for the western Atlantic and the eastern Atlantic Ocean south of the Walvis Ridge with data points indicated by large black dots^15^,^16^,^55^. (**b**) Map showing location of seawater *ɛ*_Nd_ profiles. (**c**) Reconstruction of Holocene Atlantic *ɛ*_Nd_ with data points indicated by large black dots (data is listed in Supplementary Tables 2,3 and 4). (**d**) Map showing location of sites used in the Holocene and LGM reconstructions. Figure created using Ocean Data View software^22^.

We gridded our Holocene foraminiferal *ɛ*_Nd_ measurements (Supplementary Table 2) with suitable published data (Fig. 2c). The published data includes results from measurements made on foraminifera, fish debris, high resolution crusts and leachates (Supplementary Tables 3 and 4). As some measurements made on leachates have been shown to be susceptible to contamination by the detrital fraction^19^,^20^, results from sites where the core top leachate *ɛ*_Nd_ values deviated significantly from nearby seawater values were excluded from the reconstruction. The data points included from the crusts BM1969.05 and TR079 D-14 (Supplementary Table 4) were dated outside of the definition of the LGM used for selecting sediment core data (23–18 ka); however, the records from these crusts were used to argue for the stability of seawater *ɛ*_Nd_ across glacial–interglacial cycles in their respective locations^21^. The slightly older glacial data points from these crusts were therefore deemed appropriate for inclusion in the LGM time slice. DIVA gridding was performed using Ocean Data View^22^ with the ‘signal-to-noise ratio' variable set to a value of 25. This variable determines how much a single data point is able to influence the overall plot and this level was chosen to reduce the influence of single data points, as was done by Curry and Oppo^4^.

The Holocene reconstruction shows the most unradiogenic *ɛ*_Nd_ values, around −13, below 1,500 m at the northernmost extent of the plot (Fig. 2c). These values become more radiogenic to the south, with the greatest propagation of this unradiogenic signal centred at 2,000–4,000 m water depth. This is surrounded by areas with more radiogenic *ɛ*_Nd_ values, from −8 to −10, at all depths south of 25° S; below 4,000 m from 25° S to the equator; and above 1,500 m at all latitudes. Although the data set is not as extensive as that used in δ^13^C reconstructions^4^, this reconstruction replicates the salient features of the seawater *ɛ*_Nd_ (Fig. 2a). The only significant mismatches are above 1,000 m at all latitudes and below 4,000 m north of 40° N; in these regions the gridding procedure extrapolates from the nearest data point to the edge of the profile due to a lack of data based constraints. These areas should therefore be interpreted with caution and for this reason the depth range of 0–1,000 m is excluded from later reconstructions.

### Last Glacial Maximum Atlantic *ɛ*_Nd_ reconstruction

**Figure 3:**
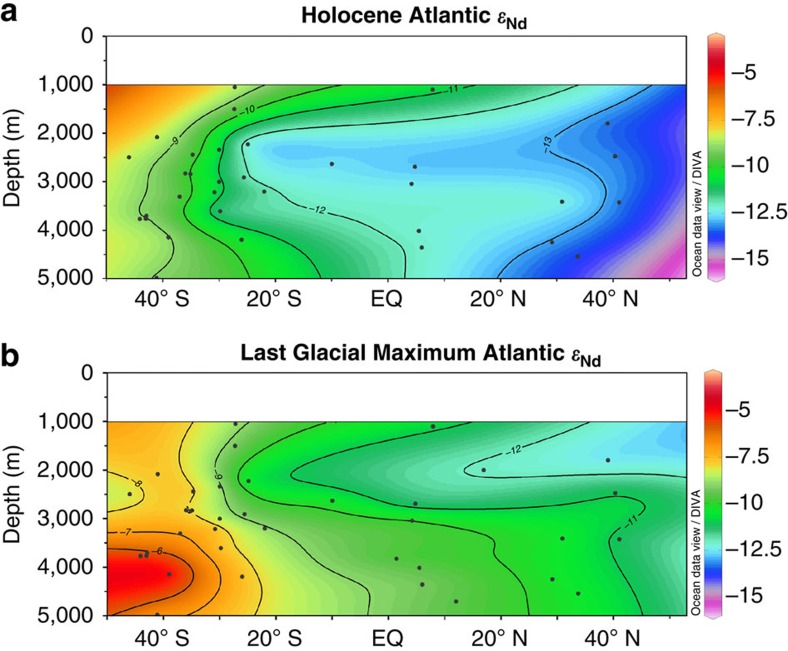
Holocene and LGM Atlantic *ɛ*_Nd_ reconstructions. (**a**) Holocene and (**b**) LGM (23–18 ka) reconstructions of *ɛ*_Nd_ of the Atlantic Ocean from 50° S to 53° N measured on uncleaned foraminifera in this study and published authigenic data (Supplementary Tables 2,3 and 4)^18^,^21^,^25^,^26^,^32^,^56^,^57^,^58^,^59^. Core sites are given by the black dots; locations are plotted in Fig. 2d and Supplementary Fig. 1. Depths from 0 to 1,000 m have been left blank due to a lack of data. Figure created using Ocean Data View software^22^.

The good correlation between seawater *ɛ*_Nd_ and the salinity profile of the modern Atlantic^23^ demonstrates that the modern seawater *ɛ*_Nd_ profile is the result of mixing between northern- and southern-sourced water masses. Collectively, these observations provide confidence that this selection of cores can be used to constrain past Atlantic water mass distributions below 1,000 m. The LGM *ɛ*_Nd_ reconstruction (Fig. 3b) shows the most unradiogenic values around −12.5 between 1,500 and 2,000 m extending from the North Atlantic to ∼10° N which then transition to values around −8 at 40° S. In contrast, the deep North Atlantic is occupied by a homogeneous water mass with an *ɛ*_Nd_ around −10.5; *ɛ*_Nd_ values become more radiogenic to the south, reaching −5.5 at 45° S. The largest differences between the LGM and modern Atlantic profiles occur below 2,500 m (Fig. 3); the deep North Atlantic shifts from *ɛ*_Nd_ values around −10.5 in the glacial to −13.5 in the modern ocean, whilst the deep South Atlantic shifts from −5.5 to −8.5. This coherent spatial pattern of changes in *ɛ*_Nd_ values in the Atlantic between the LGM and the Holocene (Fig. 3b) is consistent with a change in water mass advection and indicates there was a greater influence of southern-sourced waters in the deep Atlantic under glacial conditions.

## Discussion

The change observed in the deep South Atlantic between the LGM and Holocene (Fig. 3) is consistent with a lower flux of NADW into the Southern Ocean under cooler, glacial conditions^24^,^25^. This lower flux of NADW resulted in the deep Southern Ocean being filled with a greater proportion of water of Pacific and Indian Ocean origin that was more radiogenic in composition than Atlantic-sourced deep water^26^,^27^,^28^. The deep South Atlantic, however, remained less radiogenic than the deep Pacific^26^,^28^, requiring a source of unradiogenic neodymium to the deep South Atlantic under glacial conditions. Furthermore, the deep North Atlantic was less radiogenic than the deep South Atlantic during the LGM (Fig. 3b); this implies that northern-sourced water with its unradiogenic *ɛ*_Nd_ must have been exported to abyssal depths under glacial climate conditions. The mixing of GNAIW with denser southern-sourced waters is deemed an unlikely explanation given the large amount of energy required to mix these intermediate waters down to abyssal depths in the North Atlantic. Rather, these observations suggest that two glacial North Atlantic-sourced water masses existed during the LGM: GNAIW at depths above 2,500 m and denser Glacial NADW (GNADW)^29^ below 2,500 m.

The inference of a lower flux of GNADW under glacial conditions^24^ than NADW in the Holocene resulting in a more radiogenic composition of Glacial AABW (GAABW) than AABW would cause the deep Atlantic to exhibit more radiogenic *ɛ*_Nd_ values without a change in the water mass mixing proportions in the deep Atlantic. By taking this end-member change of deep southern-sourced water *ɛ*_Nd_ into account, however, our results can be used to calculate the proportion of the Atlantic ventilated by NADW (%NADW) both in the Holocene and during the LGM (Supplementary Table 5), and thus elucidate changes in water mass mixing proportions from the effect of the change in the AABW end-member composition. In each case three end-members were required; namely NADW; AABW and Antarctic Intermediate Water, or their glacial counterparts (Supplementary Fig. 2). A binary mixing calculation was performed between a northern- and a southern-sourced water mass at each core site using equation (1). The choice of southern-sourced water varied with depth and latitude according to the observed boundary between Antarctic Intermediate Water and Lower Circumpolar Deep Water as defined by a salinity of 34.7 psu (ref. 30) and the gradient in neodymium concentration (but not *ɛ*_Nd_) between them in the Atlantic sector of the modern Southern Ocean^16^.


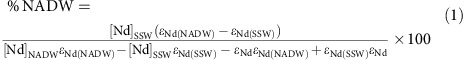


The modern seawater *ɛ*_Nd_ and [Nd] end-member values were taken from published seawater data (Supplementary Fig. 2). For the glacial ocean, the *ɛ*_Nd_ and [Nd] of NADW and PDW were kept constant; support for the stability of the *ɛ*_Nd_ of both comes from crust data^31^ whilst there is, at present, no proxy for past neodymium concentration available. The more radiogenic *ɛ*_Nd_ of GAABW was taken from a South Atlantic record of benthic foraminifera^32^ and the concentration calculated by conservative mixing of NADW and PDW (ref. 16). Unlike records from the deep South Atlantic^26^,^32^, the new intermediate depth glacial foraminiferal *ɛ*_Nd_ data from the South Atlantic measured in this work exhibit no changes between the LGM and the Holocene (GeoB2107-3 and GeoB2104-3; Supplementary Table 2), so the neodymium composition of Glacial Antarctic Intermediate Water was assumed to be the same as in the modern ocean. This observation clearly demonstrates that the intermediate and deep South Atlantic were isotopically distinct in terms of neodymium during the LGM, reinforcing the need for two distinct southern-sourced end-member water masses in the mixing calculations.

**Figure 4:**
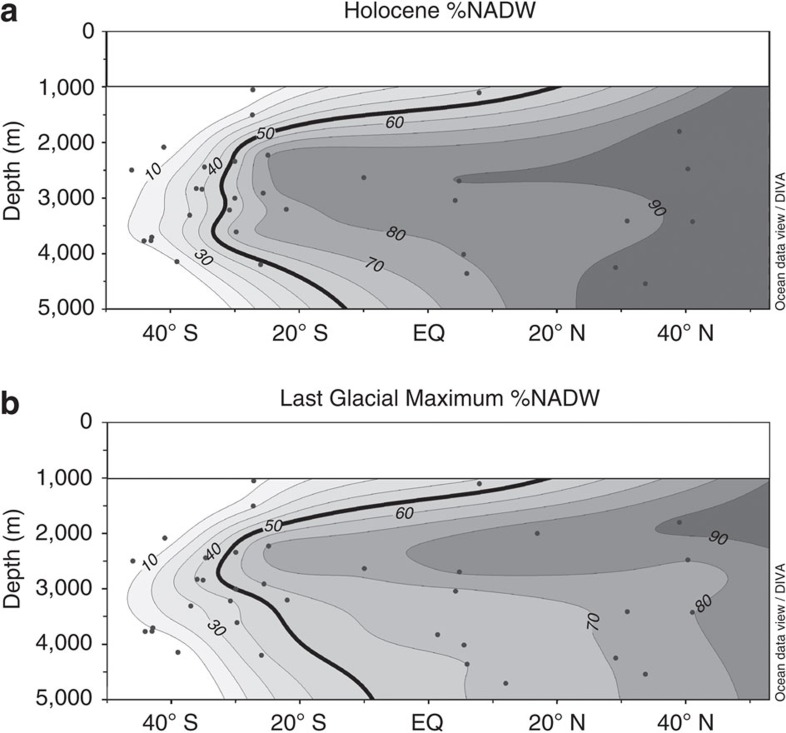
Holocene and LGM NADW percentage. NADW percentage (%NADW) calculated for the Atlantic Ocean from 50° S to 53° N in the (**a**) Holocene and during the (**b**) LGM (23–18 ka) using authigenic *ɛ*_Nd_. Depths from 0 to 1,000 m have been left blank due to a lack of data. Figure created using Ocean Data View software^22^.

Figure 4 shows the %NADW values calculated for the Holocene and LGM Atlantic gridded into meridional sections using the same technique employed for the *ɛ*_Nd_ data profiles plotted in Fig. 2. In the LGM *ɛ*_Nd_ profile (Fig. 3b), the most radiogenic values, around −5.5, are observed at 4,000 m depth in the South Atlantic, whereas less radiogenic values, around −6.5, are seen at ∼5,000 m at the same latitude. The more radiogenic values come from the Mid-Atlantic Ridge; the less radiogenic from the Cape Basin, which lies to the east of the Mid-Atlantic Ridge. The *ɛ*_Nd_ difference between these sites likely reflects a longitudinal mixing gradient between radiogenic, Pacific-derived, circumpolar waters^28^ and less radiogenic North Atlantic derived waters^25^. A similar phenomenon is seen in modern seawater data from the South Atlantic although the offset is less pronounced^16^. The results from the deep Cape Basin^26^ were, therefore, excluded from the mixing proportion plots in Fig. 4 as the longitudinal gradient in *ɛ*_Nd_ observed in the deep South Atlantic obscures the latitudinal mixing gradient which is the primary interest here.

The gridded profile of the Holocene %NADW results (Fig. 4a) shows the Atlantic north of 20° N and below 1,500 m depth is mostly (>90%) NADW. %NADW decreases at all depths to the south, with the 50% mixing line occurring near 10° S at 5,000 m, but not until 30° S from 2,000 to 4,000 m. The LGM %NADW contours, including the 50% contour, (Fig. 4b) are similar to the Holocene profile above 2,500 m (Fig. 4a). The greatest difference between the two plots occurs below 2,500 m in the North Atlantic, which has significantly less NADW in the LGM profile (55–80% NADW) than in the Holocene profile (>90% NADW). From this observation it is clear that there was a greater proportion of southern-sourced waters in the deep Atlantic during the LGM than in the Holocene; however, it is also clear that the deep Atlantic was not occupied solely by southern-sourced waters during the LGM.

**Figure 5:**
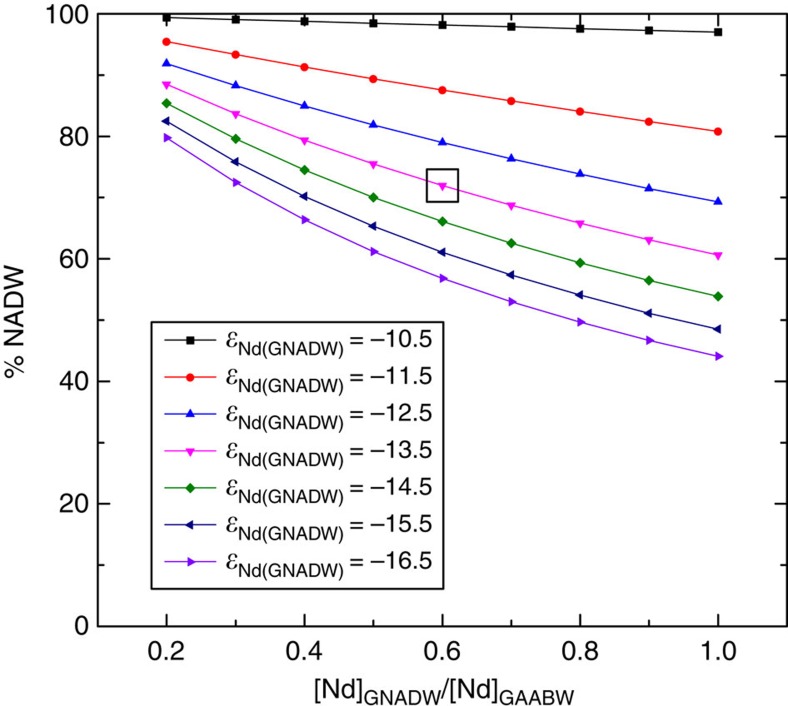
Sensitivity test of NADW percentage calculation. Results of the sensitivity tests performed on the %NADW calculated for the Bermuda Rise *ɛ*_Nd_ of −10.4 during the LGM (OCE326-GGC6; 33.7° N, 57.6° W, 4,540 m)^18^. *ɛ*_Nd(GAABW)_ was held constant at −5.5 (ref. 32), whilst *ɛ*_Nd(GNADW)_ and the ratio of [Nd]_GNADW_ to [Nd]_GAABW_ were varied as shown. The result from end-members used in Fig. 4 (*ɛ*_Nd(GNADW)_=−13.5 and [Nd]_GNADW_/[Nd]_GAABW_=0.60) is highlighted by a black box.

Although changes in the proportion of NADW in the deep Atlantic between glacial and interglacial conditions are the simplest explanation for the results in Figs 3 and 4, other alternatives must be considered. The sensitivity of the calculated %NADW for the glacial deep Bermuda Rise *ɛ*_Nd_ of −10.4 (OCE326-GGC6; 33.7° N, 57.6° W, 4,540 m)^18^ to the neodymium composition of end-member water masses is shown in Fig. 5. The *ɛ*_Nd_ of AABW, considered to be known with reasonable confidence^32^, was held constant. The *ɛ*_Nd_ of GNADW was varied from a near maximum possible value of −10.5 to a minimum of −16.5, similar to the least radiogenic value of −16 that was used in similar calculations performed for a nearby site^33^. As the %NADW result is dependent on the relative neodymium concentrations of the northern and southern end-members, the ratio of [Nd]_GNADW_ to [Nd]_GAABW_ was varied from 0.2 to 1. The latter value was used in similar calculations performed for a South Atlantic site^32^ and is used as an upper limit in this work as ratios >1 would require more neodymium dissolved in the deep Atlantic than the deep Pacific, a situation that is not consistent with the observation that neodymium concentrations increase with water mass age in the modern ocean^34^.

Using the glacial end-member values assigned in Fig. 4, the deep Bermuda Rise site was calculated to be bathed by 72% NADW during the LGM (black box, Fig. 5). In contrast an *ɛ*_Nd(GNADW)_ of −10.5 and concentration ratio of 0.2 yields nearly 100% NADW, whilst an *ɛ*_Nd(GNADW)_ of −16.5 and a concentration ratio of 1.0 means just 44% NADW is required to explain the *ɛ*_Nd_ observed at the Bermuda Rise during the LGM. Although the resultant uncertainty in the %NADW at the Bermuda Rise of ±28% is significant, all end-member configurations yield %NADW in the glacial deep North Atlantic values >44%. This is a robust finding that directly contradicts the notion that the deep Atlantic was dominated by southern-sourced waters during the LGM. Furthermore, the lower estimate of 44% is deemed unlikely to be realistic as less radiogenic *ɛ*_Nd(NADW)_ values appear restricted to interstadials, and have been interpreted as pulses of Labrador Sea Water formation during these warm intervals^33^ or unradiogenic weathering pulses during ice sheet retreat^35^. Rather, most evidence suggests that *ɛ*_Nd(GNADW)_ values were similar to or slightly more radiogenic than NADW in the modern Atlantic^21^ yielding higher %NADW values (Fig. 5). The influence of analytical error on the calculated %NADW values is small; using the external error bounds of 0.5 epsilon units^18^ gives a range of 67–77% NADW at the deep Bermuda Rise during the LGM.

Although these uncertainties do limit the certainty of the exact proportion of the deep Atlantic that was ventilated by northern-sourced waters during the LGM, the need for Glacial NADW appears inescapable. If localized processes, such as boundary exchange, were controlling the glacial *ɛ*_Nd_ profile, one would expect to see a heterogeneous profile controlled by regional detrital inputs. Furthermore, modelling studies have shown that boundary exchange processes are not able to explain the magnitude of the observed glacial–interglacial shifts in deep ocean *ɛ*_Nd_ and thus changes in water mass advection must be invoked to explain them^36^. Reconstructions of glacial deep water mass ventilation from radiocarbon^37^, and carbonate ion concentration from B/Ca^38^,^39^ also show a younger, better ventilated, water mass in the deep North Atlantic than the deep South Atlantic. These proxies, therefore, also provide evidence for a significant proportion of northern-sourced deep waters in the deep North Atlantic during the LGM, supporting the results shown in Fig. 4.

**Figure 6:**
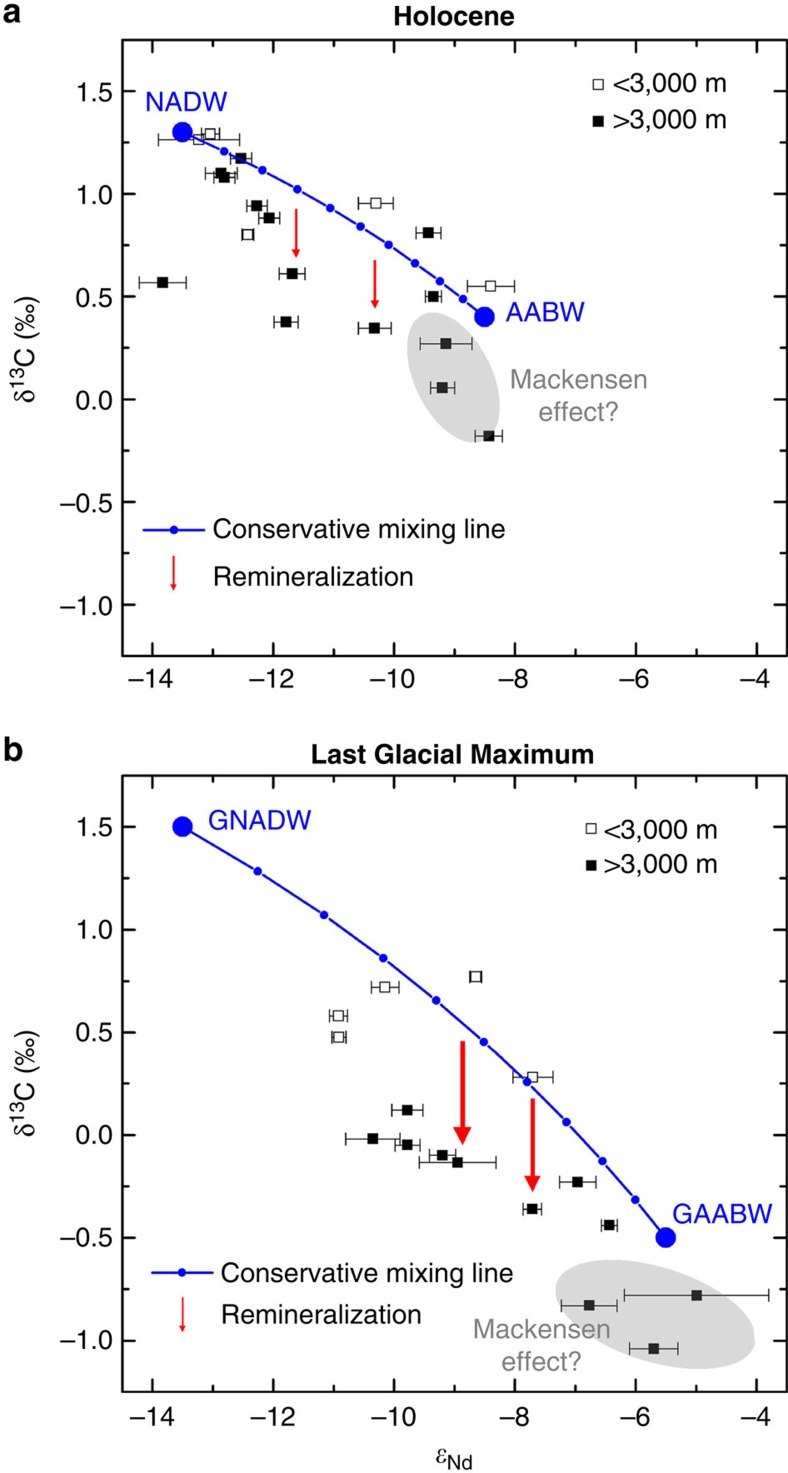
Cross plots of Atlantic benthic foraminiferal δ^13^C against foraminiferal *ɛ*_Nd_. Cross plots of benthic foraminiferal δ^13^C against foraminiferal *ɛ*_Nd_ for the deep Atlantic Ocean during the (**a**) Holocene and the (**b**) LGM. Water mass end-members labelled are NADW, AABW, GNADW and GAABW. The blue curve shows the values expected for conservative mixing between these water masses in the corresponding cross plot. Offsets from this line are attributed to either the remineralization of organic matter (red arrows) or a possible Mackensen Effect (shaded grey regions)^40^. Details of the data used are given in Supplementary Table 7, and how end-member values were assigned in Supplementary Note 1 and Supplementary Table 8. Error bars show the 2σ external error of the *ɛ*_Nd_ measurements.

Reconciling the glacial *ɛ*_Nd_ values in the Atlantic with nutrient proxy based reconstructions requires a greater amount of respired organic carbon in the deep glacial Atlantic relative to the modern situation^4^,^8^. Circulation versus biological sources of carbon can be differentiated by cross plots of *ɛ*_Nd_ against benthic foraminiferal δ^13^C for the Holocene and LGM Atlantic (Fig. 6). Deep water mass mixing end-members were ascribed to each plot as detailed in the Supplementary Information. The South Atlantic foraminiferal values (grey regions Fig. 6) were corrected for the Mackensen Effect, the phenomenon where benthic foraminifera display δ^13^C values lower than that of the overlying deep water due to a phytodetrital layer of light organic carbon on the sea floor^40^. The composition of NADW is similar in the Holocene and LGM cross plots with δ^13^C and ɛ_Nd_ values ∼1.4‰ and −13.5, respectively, whereas glacial AABW δ^13^C is more depleted (−0.5‰) and *ɛ*_Nd_ more radiogenic (−5.5) than its modern counterpart (0.4‰, −8.5).

The blue curves show the values expected for conservative mixing between these end-members for each time slice; those points falling along this mixing line have seen little input of biological carbon. During the LGM, data points lying close to the mixing line come from cores shallower than 3,000 m. Most of the glacial data points from cores located below 3,000 m sit well below the mixing curve which we interpret as being caused by the addition of respired organic matter with a low δ^13^C (usually∼−20‰)^41^. It is important to note that these cores represent locations within both northern- and southern- sourced glacial deep water, so this signal was not advected from either source but was acquired by biological processes along the advection pathway; this highlights the shortcomings of using δ^13^C in isolation as a proxy for water mass mixing. The lower than expected δ^13^C values could also in part be due to the use of mixed *Cibicidoides* species for some of the benthic foraminiferal δ^13^C measurements. The variable depth habitats of different *Cibicidoides* species could lead to offsets of the measured foraminiferal calcite δ^13^C from bottom water δ^13^C (ref. 42). The inference of organic matter remineralization being the dominant cause of the offset, however, is supported by a recent study that modelled the glacial Atlantic and predicted a biological regenerative imprint in the glacial deep Atlantic δ^13^C (ref. 8). The same study calculated that, after allowing for this remineralisation of organic matter, the glacial deep North Atlantic was ventilated by 50–80% NADW, which compares well with the *ɛ*_Nd_ derived values of 55–80% NADW produced in this work (Fig. 4b).

The accumulation of respired organic carbon in the deep Atlantic during the LGM could be due to higher surface productivity and thus export of organic carbon to the deep ocean under glacial climate conditions. Although glacial productivity reconstructions in the northwestern Atlantic are sparse, nearby regions show elevated export production during the LGM relative to the Holocene^43^. Higher glacial surface productivity, however, cannot explain the depth dependence of the δ^13^C offset in the LGM (Fig. 6b), nor can it explain the chemocline seen at ∼2,500 m in nutrient proxy reconstructions^3^,^4^. Thus it seems likely that the greater amount of respired organic matter in the deep Atlantic during the LGM must have also been at least partially due to a longer residence time of seawater in the glacial deep Atlantic than in the modern Atlantic. This conclusion is supported by a modelling study of a ^231^Pa/^230^Th data compilation which concluded that the glacial Atlantic had rapid overturning in the shallow cell but slower overturning at depth^44^.

**Figure 7:**
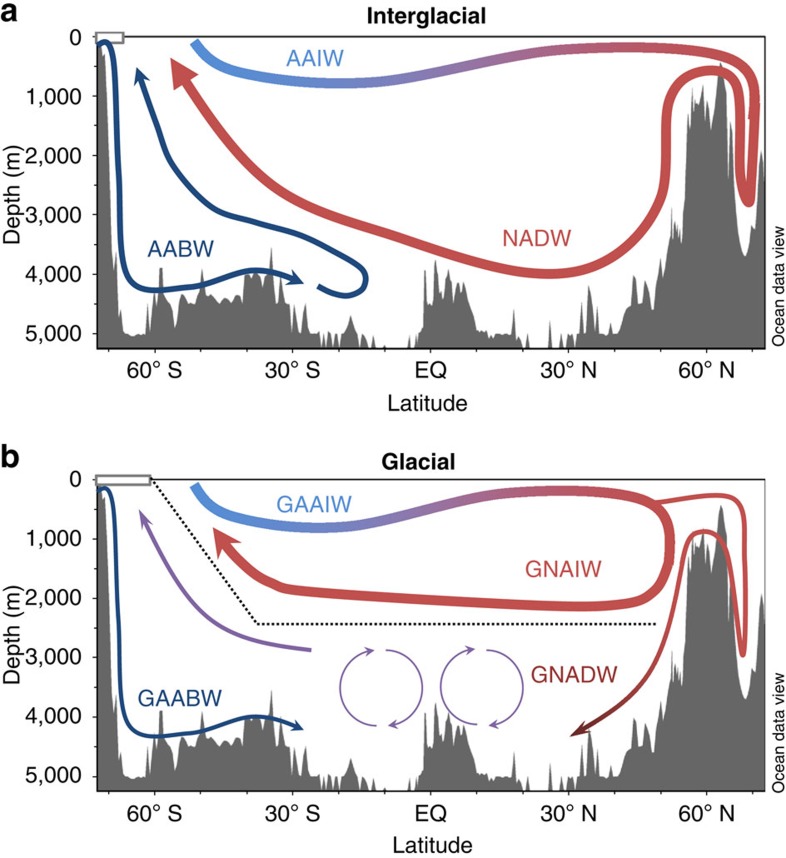
Interglacial and Glacial Atlantic overturning schematics. Schematics of the structure of Atlantic Meridional Overturning Circulation during (**a**) interglacial and (**b**) glacial periods assuming the Holocene and LGM are typical of each. Water masses depicted are Antarctic Intermediate Water (AAIW), NADW and AABW and their glacial counterparts as well as GNAIW. Thin arrows associated with GNADW and GAABW in the glacial schematic represent the inferred lower flux of these water masses during glacial periods relative to interglacials. The dotted black line in the glacial schematic shows the location of the chemocline in nutrient proxy reconstructions at the interface between the two overturning cells. Figure partly created using Ocean Data View software^22^.

Our findings are summarized in hypothetical overturning schematics for the Atlantic Ocean during glacials and interglacials in Fig. 7. In the glacial scenario (Fig. 7b) there are two distinct northern-sourced water masses, separated by density differences. The glacial northern-sourced intermediate depth water mass was likely formed by convection south of Iceland^45^ and is inferred to have overturned rapidly^44^. The deep Atlantic cell is surmised to have overturned more slowly and may have been supplied by NADW formed in the Nordic Seas during seasonal ice-free periods coming over the Greenland-Scotland Ridge^46^. Brine rejection from sea ice may also have contributed to this process^47^. A lower flux of GNADW during the LGM than NADW in the Holocene in to the deep North Atlantic would then explain how AABW was able to penetrate much further north during the LGM (Fig. 4b) than in the modern Atlantic Ocean where it has little influence north of the equator (Fig. 4a). In contrast, the deep South Atlantic shows less of a change in water mass mixing proportions between the LGM and the Holocene (Fig. 4) because it is dominated by southern-sourced waters in both climate states (Fig. 7).

Many modelling studies and hypothesized glacial overturning schemes invoke GNAIW being incorporated into deep waters elsewhere in the glacial ocean to ventilate the deep ocean with low-preformed nutrient concentration water^10^,^13^,^48^. Here, we have presented evidence that instead the deep Atlantic was ventilated directly from the North Atlantic resolving the difficulty in reconciling glacial CO_2_ drawdown with the observed proxy data. Another important prediction of our work is that switching from the glacial to interglacial mode of North Atlantic circulation (Fig. 7) would flush respired organic carbon from the deep Atlantic and would therefore be expected to raise atmospheric CO_2_ without invoking changes in nutrient utilization in the high latitude Southern Ocean^13^. Although a full carbon cycle model would be required to quantify this effect, the resumption of strong NADW production at the start of the Bølling-Allerød inferred from *ɛ*_Nd_ and ^231^Pa/^230^Th records, coincides with a ∼12 p.p.m. increase in atmospheric CO_2_ (Supplementary Fig. 3)^12^,^18^,^49^. This increase likely reflects the flushing of respired carbon from the deep Atlantic by strong NADW production during the deglaciation^50^.

## Methods

### Site selection

*ɛ*_Nd_ measurements were made on uncleaned planktic foraminifera from Holocene and LGM samples of cores from throughout the western Atlantic basin or selected regions within the eastern Atlantic (Fig. 2d); all core names and locations are listed in Supplementary Table 1 and plotted in Supplementary Fig. 1. Eastern Atlantic cores were limited to sites south of the Walvis Ridge or sites on bathymetric rises above the sill depth of the Mid-Atlantic Ridge (3,750 m)^51^. This selection was based on the criteria outlined by Curry and Oppo^4^, and was intended to avoid the regions of the eastern Atlantic which are ventilated through fracture zones in the Mid-Atlantic Ridge and thus do not display the same latitudinal water mass mixing gradient as the western Atlantic^52^. Cores north of 45° N were excluded as they have been shown to be susceptible to the influence of volcanic ash and IRD in the North Atlantic^19^,^53^.

### Age controls

Age controls for cores used in this work range from planktic or benthic foraminiferal δ^18^O records to radiocarbon dates (Supplementary Table 1) and came from published age models, with the exception of the Ceara Rise cores. The radiocarbon-based age models developed in this work for the latter cores, ODP 925E, ODP 928B and ODP 929B, are presented in Supplementary Table 6. For all cores, depths which were assigned calendar ages between 23 and 18 ka were included in the LGM reconstruction.

### Sample preparation

Samples were prepared for analysis following the methods of Roberts *et al*.^18^ and references therein. In short, where possible, ∼80 mg of mixed planktic foraminifera were picked from the coarse fraction (>63 μm) for neodymium isotope measurements. After picking, foraminifera tests were broken open between two glass plates, rinsed, sonicated and any clays removed. The samples were then dissolved in 1 mol l^−1^ reagent grade acetic acid. The REEs were extracted from the dissolved sample using Eichrom TRUspec resin in 100 ml Teflon columns. Neodymium was then separated from the other rare earth elements using Eichrom LNspec resin on volumetrically calibrated Teflon columns.

### Neodymium isotopic measurements

Neodymium isotopes were analysed using the Nu Plasma HR or Neptune Plus multi-collector inductively coupled plasma mass spectrometers at the University of Cambridge. ^146^Nd/^144^Nd was normalized to 0.7219 and samples were bracketed with a concentration-matched solution of reference standard JNdi-1, the measured composition of which varied between runs but was corrected to the accepted value of ^143^Nd/^144^Nd=0.512115 (ref. 54). The *ɛ*_Nd_ of each sample is reported with the external error (2σ) of the bracketing standards from the corresponding measurement session, unless the internal error was larger than the external error, in which case the combined internal and external error (2σ) is reported.

Eight complete procedural blanks for the process from foraminiferal dissolution through to column chemistry were run on a TIMS Sector 54 at the University of Cambridge using a ^150^Nd spike. For the typical sample, of at least 15 ng, the average blank of 66 pg of neodymium is <0.5% of the total neodymium.

### Benthic foraminiferal stable isotopes

Benthic foraminifera (mixed *Cibicidoides* species; between 3 and 7 tests) were picked for stable isotope analysis from the coarse fraction (>125 μm) of samples without published δ^13^C data. Samples were analysed by the Godwin Laboratory at the University of Cambridge using either a Micromass Multicarb Sample Preparation System attached to a VG SIRA or a Thermo Kiel device attached to a Thermo MAT253 Mass Spectrometer in dual inlet mode. Isotopic ratios are presented relative to standard Vienna PeeDee Belemnite; external precision was ±0.06‰ for δ^13^C.

### Data availability

The data reported in this paper are listed in the Supplementary Information and archived in Pangaea (https://doi.pangaea.de/10.1594/PANGAEA.859580).

## Additional information

**How to cite this article**: Howe, J. N. W. *et al*. North Atlantic Deep Water Production during the Last Glacial Maximum. *Nat. Commun.* 7:11765 doi: 10.1038/ncomms11765 (2016).

## Supplementary Material

Supplementary Information Supplementary Figures 1-3, Supplementary Tables 1-8, Supplementary Note 1 and Supplementary References
