# Supplementary material for: North Atlantic Deep Water Production during the Last Glacial Maximum
Source: Nat Commun. 2016 Jun 3;7:11765. doi: 10.1038/ncomms11765 (PMC4895795; doi:10.1038/ncomms11765)
Supplement: Supplementary Information — Supplementary Figures 1-3, Supplementary Tables 1-8, Supplementary Note 1 and Supplementary References [file ncomms11765-s1.pdf]

Supplementary Information

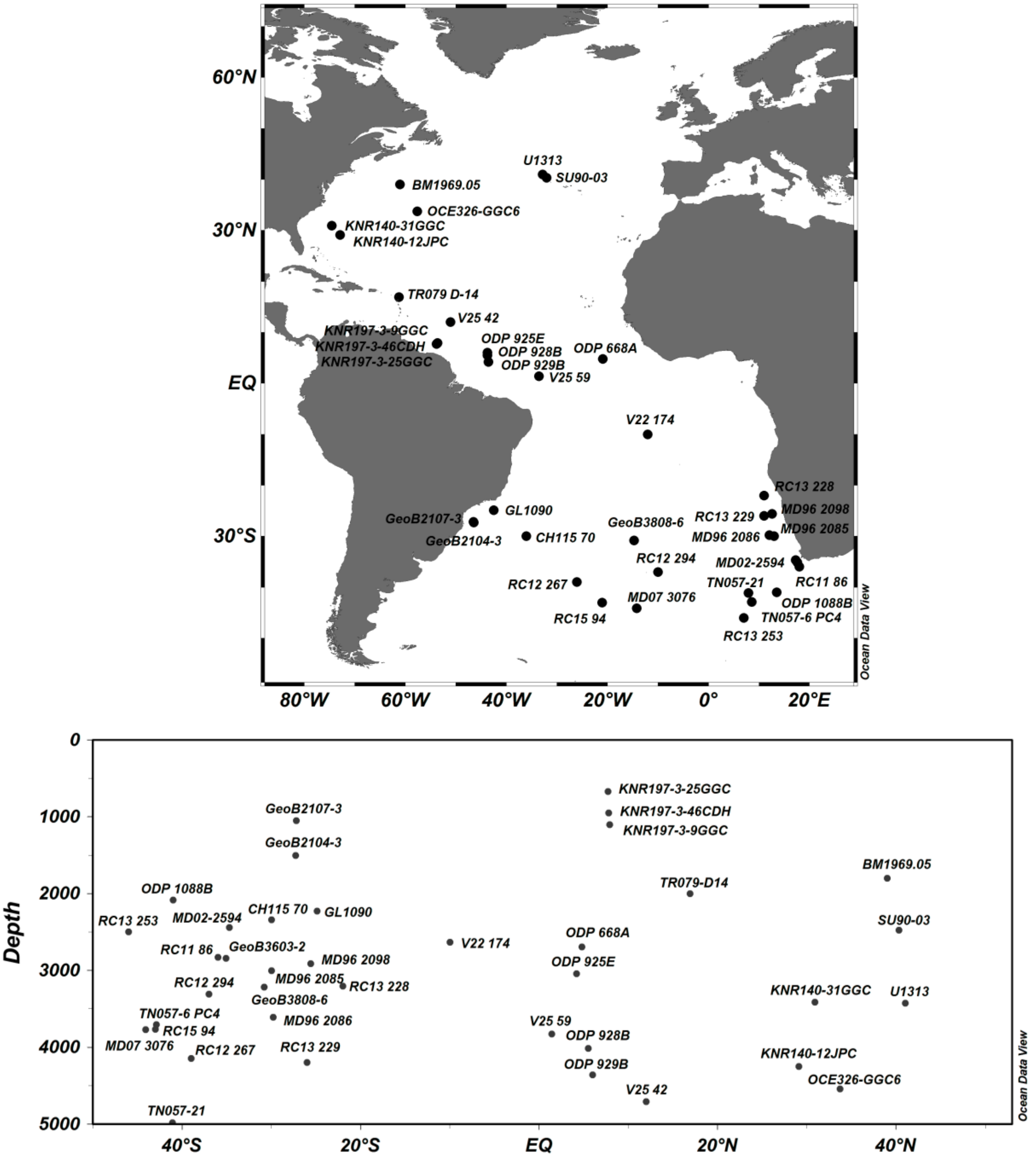

Supplementary Fig. 1 | Map and profile view of core locations. Map (top) and profile view (bottom) of the Atlantic Ocean showing the location of core sites, with names, from which data is presented in this study (Schlitzer, R. Ocean Data View. (2016)). References for the data from each core site are given in Supplementary Tables 1, 2, 3 and 4.

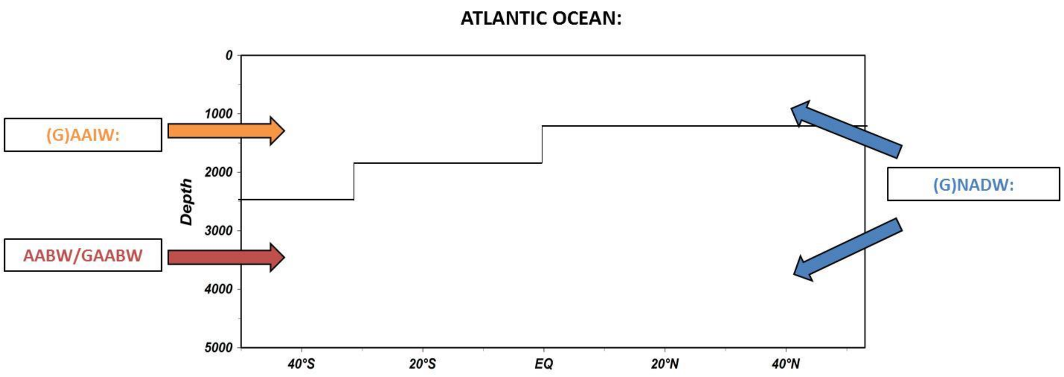

Water Mass	ε_{Nd}	[Nd] (pmol kg^{-1})	Reference
(G)NADW	-13.5	17.5	[1,2]
(G)AAIW	-8.3	13	[3]
AABW	-8.5	25.1	[3]
GAABW	-5.5	29	[4]/calculated
(G)PDW	-3.5	35	[5,6]

Supplementary Fig. 2 | End-members used in %NADW calculations. Schematic showing the division of the Atlantic Ocean into binary mixing regions according to the southern-sourced water mass used in the calculation of %NADW. Water masses labeled are (Glacial) North Atlantic Deep Water [(G)NADW], (Glacial) Antarctic Intermediate Water [(G)AAIW], (Glacial) Antarctic Bottom Water [(G)AABW] and (Glacial) Pacific Deep Water [(G)PDW]. The black line denotes the separation of (Glacial) Antarctic Intermediate Water and (Glacial) Antarctic Bottom Water and approximately follows the location of the salinity contour of 34.7 psu in the modern Atlantic[7]. End-member composition values used for %NADW calculations are listed in the table below. The [Nd] of Glacial Antarctic Bottom Water was calculated as a conservative mixture of Glacial North Atlantic Deep Water and Glacial Pacific Deep Water using ε_{Nd}.

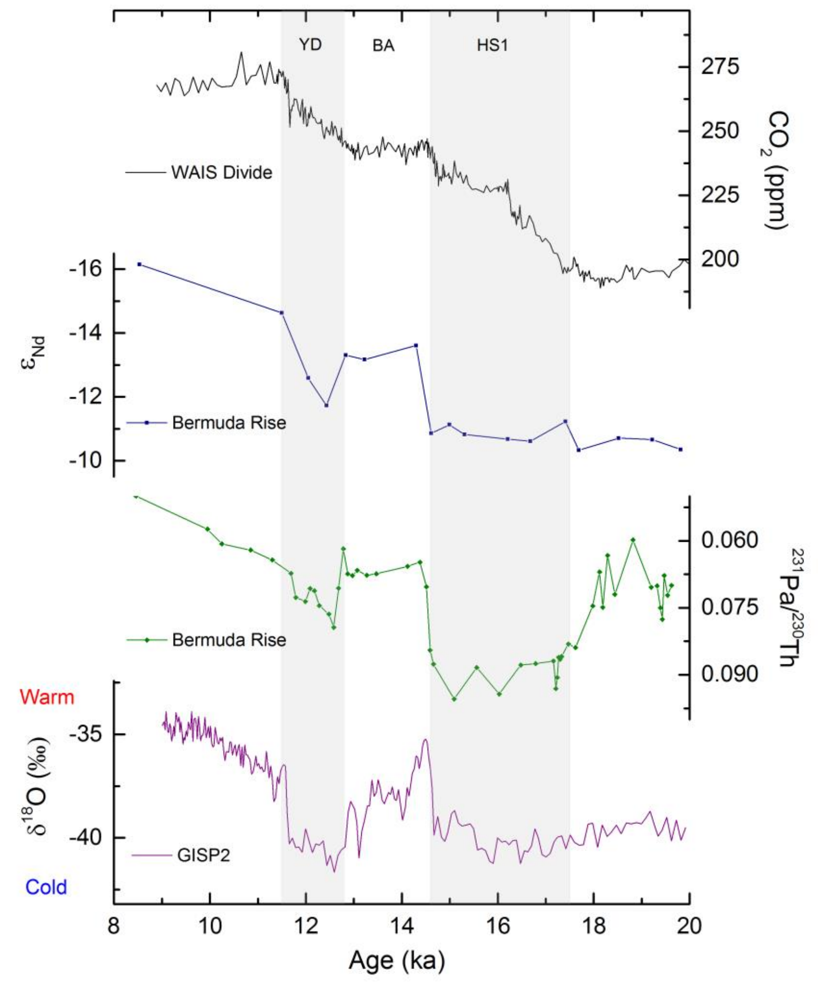

Supplementary Fig. 3 | Paleo-reconstructions of the most recent deglaciation. Deglacial records of atmospheric CO_2 from WAIS Divide[8], ε_{Nd} (OCE326-GGC6; 33.7°N, 57.6°W, 4541 m)[9] and $^{231}Pa/^{230}Th$ (OCE326-GGC5; 33.7°N, 57.6°W, 4550 m)[10] from the Bermuda Rise and $\delta^{18}O$ of ice, a proxy for Greenland temperature, from GISP2[11]. Northern Hemisphere climate periods labelled are the Younger Dryas (YD), Bølling-Allerød (B-A) and Heinrich Stadial 1 (HS1).

Supplementary Table 1. Cores used in this work to measure foraminiferal ε_{Nd} with reference to age controls and the respective studies.

Core	Lat. (°N)	Long. (°E)	Depth (m)	Age control	Reference
CH115 70	-30.0	-36.0	2340	Benthic $\delta^{18}O$	[12]
GeoB2104-3	-27.3	-46.4	1503	Planktic ^{14}C	[13]
GeoB2107-3	-27.2	-46.5	1048	Planktic ^{14}C	[14]
GL1090	-24.9	-42.5	2225	Planktic ^{14}C benthic $\delta^{18}O$	dos Santos and Chiessi, *pers. comm.*
MD96 2085	-30.0	13.0	3001	Planktic $\delta^{18}O$	[15]
MD96 2086	-29.8	12.1	3606	Benthic $\delta^{18}O$	[16]
MD96 2098	-25.6	12.6	2910	Planktic/Bulk ^{14}C	[17]
ODP 668A	4.8	-20.9	2693	Planktic $\delta^{18}O$	[18]
ODP 925E	4.2	-43.5	3041	Planktic ^{14}C Benthic $\delta^{18}O$	This study
ODP 928B	5.5	-43.7	4011	Planktic ^{14}C Benthic $\delta^{18}O$	This study
ODP 929B	6.0	-43.7	4356	Planktic ^{14}C Benthic $\delta^{18}O$	This study
ODP 1088	-41.0	13.5	2082	Benthic $\delta^{18}O$	[19]
RC11 86	-36.0	18.0	2829	Planktic $\delta^{18}O$	[20]
RC12 267	-39.0	-26.0	4144	Planktic $\delta^{18}O$	[21]
RC12 294	-37.0	-10.0	3308	Planktic $\delta^{18}O$	[21]
RC13 228	-22.0	11.0	3204	Benthic $\delta^{18}O$	[22]
RC13 229	-26.0	11.0	4194	Benthic $\delta^{18}O$	[23]
RC13 253	-46.0	7.0	2494	Planktic $\delta^{18}O$	[21]
RC15 94	-43.0	-21.0	3762	Planktic $\delta^{18}O$	[21]
SU90-03	40.3	-32.0	2475	Planktic $\delta^{18}O$ benthic $\delta^{18}O$	[24]
TTN057-6 PC4	-42.9	8.6	3702	Planktic $\delta^{18}O$ benthic $\delta^{18}O$	[25]
V22 174	-10.0	-12.0	2630	Planktic $\delta^{18}O$ Planktic ^{14}C	[26]
V25 42	12.0	-51.0	4707	%$CaCO_3$ tied to ODP 929	[27]
V25 59	1.4	-33.5	3824	Carbonate ^{14}C Planktic $\delta^{18}O$	[28,29]

Supplementary Table 2. Holocene and LGM ε_{Nd} measured on planktic foraminifera in this work. All samples were 1 cm in thickness; for cores with multiple LGM measurements the mean value was taken.

**Core**	**Holocene**			**Last Glacial Maximum**		
	**Depth (cm)**	**ε_{Nd}**	**2σ**	**Top depth (cm)**	**ε_{Nd}**	**2σ**
CH115 70	0	-10.3	0.3	30	-8.6	0.1
GeoB2104-3	5	-9.3	0.2	139	-9.2	0.2
GeoB2107-3	9	-8.5	0.2	140	-8.8	0.1
GL1090	0	-13.0	0.2	54, 58, 60	-10.9	0.1
MD96 2085	20	-10.6	0.2	110	-8.6	0.3
MD96 2086	42	-11.8	0.2	159	-7.8	0.2
MD96 2098	25	-12.2	0.2	231	-9.3	0.2
ODP 668A	8	-12.9	0.1	20	-11.6	0.1
ODP 925E	8	-12.5	0.2	68	-9.8	0.3
ODP 928B	8	-12.3	0.2	58, 68	-9.5	0.3
ODP 929B	3	-12.1	0.2	53, 58, 63	-9.2	0.2
ODP 1088	0	-8.4	0.4	34	-7.7	0.3
RC11 86	1	-9.9	0.2	40	-8.0	0.1
RC12 267	2	-8.8	0.8	70	-4.9	0.2
RC12 294	9	-9.4	0.2	50	-7.0	0.3
RC13 228	2.5	-11.7	0.2	153	-8.9	0.6
RC13 229	10	-10.3	0.3	80	-7.7	0.2
RC13 253	20	-8.5	0.3	40	-8.3	0.3
RC15 94	8	-8.4	0.2	90	-5.0	1.2
SU90-03	1	-13.2	0.7	85, 92, 95, 100	-10.9	0.1
TTN057-6 PC4	39	-9.4	0.1	75	-6.4	0.1
V22 174	10	-12.4	0.1	60	-10.1	0.2
V25 42	-	-	-	35	-9.6	0.4
V25 59	-	-	-	56	-9.8	0.2

Supplementary Table 3. Published core data used in constructing Holocene and LGM profiles

Core	Lat. (°N)	Long. (°E)	Depth (m)	Holocene			Last Glacial Maximum			Ref.
				Depth (cm)	ε_{Nd}	2σ	Depth (cm)	ε_{Nd}	2σ	
GeoB3603-2	-35.1	17.6	2840	5	-10.1	0.2	32, 37	-8.0	0.3	[30]
GeoB3808-6	-30.8	-14.7	3213	2	-10.7	0.4	14	-7.5	0.3	[31]
KNR140-12JPC	29.1	-72.9	4250	20	-13.0	0.2	220, 233, 251, 263	-10.3	0.7	[32]
KNR140-31GGC	30.9	-74.5	3410	0	-12.2	0.5	330, 410, 420	-10.3	0.3	[32]
KNR197-3-25GGC	7.7	-53.8	671	32.5	-10.2	0.3	233	-10.3	0.3	[33]
KNR197-3-46CDH	7.8	-53.7	947	0	-10.5	0.3	321	-10.5	0.3	[33]
KNR197-3-9GGC	7.9	-53.6	1100	0.5	-10.8	0.3	349	-10.9	0.3	[33]
MD02-2594	-34.7	17.3	2440	98	-9.7	0.3	283, 297	-7.8	0.4	[30]
MD07 3076	-44.1	-14.2	3770	3	-9.2	0.2	145	-5.7	0.4	[4]
OCE326-GGC6	33.7	-57.6	4540	4	-13.8	0.4	455	-10.4	0.5	[9]
TNO57-21	-41.1	7.9	4981	95	-9.1	0.4	240	-6.8	0.5	[34]
U1313	41.0	-32.8	3426		-13.4	0.3		-11.3	0.8	[35]

Supplementary Table 4. Published high resolution crust data used in constructing Holocene and LGM profiles

Crust	Lat. (°N)	Long. (°E)	Depth (m)	"Holocene" ε_{Nd}	2σ	Age (ka)	"Glacial" ε_{Nd}	2σ	Age (ka)	Ref.
TR079 D-14	16.9	-61.2	2000	-	-	-	-12.1	0.7	29	[2]
BM1969.05	39	-61	1800	-13.38	0.67	13	-12.6	0.5	48	[2]

Supplementary Table 5. %NADW calculated for Holocene and LGM ε_{Nd} values

**Core**	**Holocene (%NADW)**	**LGM (%NADW)**
BM1969.05	99	93
CH115 70	45	52
GeoB2104-3	14	14
GeoB2107-3	3	8
GeoB3603-2	40	43
GeoB3808-6	52	35
GL1090	93	78
KNR140-12JPC	93	71
KNR140-31GGC	80	71
KNR197-3-25GGC	30	32
KNR197-3-46CDH	35	35
KNR197-3-9GGC	41	43
MD02-2594	32	40
MD07 3076	19	4
MD96 2085	50	52
MD96 2086	73	40
MD96 2098	81	60
OCE326-GGC6	100	72
ODP 668A	92	84
ODP 925E	86	66
ODP 928B	81	62
ODP 929B	78	59
ODP 1088	0	0
RC11 86	37	43
RC12 267	8	0
RC12 294	25	27
RC13 228	72	56
RC13 229	45	39
RC13 253	0	0
RC15 94	0	0
SU90-03	96	78
TNO57-21	17	24
TR079 D-14	-	88
TTN057-6 PC4	23	18
U1313	99	81
V22 174	84	70
V25 42	-	64
V25 59	-	66

Supplementary Table 6. Age models for cores 925, 928 and 929.

	Depth (cm)	ID	^{14}C Age (yrs)	Sds (σ)	Species	Calibration Curve
**ODP 925E**						
1	8-10	SUERC-53665	3835	37	*G. sacculifer*	Marine13
2	21-22	SUERC-53666	8572	38	*G. sacculifer*	Marine13
3	34-35	SUERC-53667	11099	40	*G. sacculifer*	Marine13
4	48-50	SUERC-53668	12374	41	*G. sacculifer*	Marine13
5	58-60	SUERC-53669	13120	42	*G. sacculifer*	Marine13
6	78-80	SUERC-53670	19390	68	*G. sacculifer*	Marine13
7	118-120	SUERC-53671	27650	161	*G. sacculifer*	Marine13
**ODP 928B**						
1	8-10	SUERC-53674	5363	37	*G. sacculifer*	Marine13
2	28-30	SUERC-53718	10545	39	*G. sacculifer*	Marine13
3	58-60	SUERC-53675	16159	51	*G. sacculifer*	Marine13
4	108-110	SUERC-53676	25191	122	*G. sacculifer*	Marine13
**ODP 929B**						
1	3-5	SUERC-53678	5211	37	*G. sacculifer*	Marine13
2	13-15	SUERC-53679	7870	36	*G. sacculifer*	Marine13
3	23-25	SUERC-53680	10604	38	*G. sacculifer*	Marine13
4	33-35	SUERC-53681	12457	41	*G. sacculifer*	Marine13
5	53-55	SUERC-53684	16138	52	*G. sacculifer*	Marine13
6	83-85	SUERC-53685	21673	84	*G. sacculifer*	Marine13
7	113-115	SUERC-53686	27296	156	*G. sacculifer*	Marine13

Supplementary Table 7. Cores, data and sources for benthic foraminiferal $\delta^{13}C$ used in cross plots

**Core**	**Holocene $\delta^{13}C$ (‰)**	**LGM $\delta^{13}C$ (‰)**	**Reference**
CH115 70	0.95	0.77	[12]
GL1090	1.29	0.58	dos Santos and Chiessi, *pers. comm.*
MD07 3076	0.06	-1.04	[36]
MD96 2086	0.38	-	This work
OCE326-GGC6	0.57	-0.02	[37]
ODP 925E	1.17	0.12	This work
ODP 928B	0.94	-	This work
ODP 929B	0.88	-0.10	This work
ODP 1088	0.55	0.28	[19,25]
RC12 294	0.81	-0.23	[38]
RC13 228	0.61	-0.14	[22]
RC13 229	0.34	-0.36	[23]
RC15 94	-0.18	-0.78	[39]
SU90-03	1.26	0.48	[24]
TTN057-6 PC4	0.50	-0.44	[25]
TNO57-21	0.27	-0.83	[39]
V22 174	0.80	0.72	[38]

Supplementary Table 8: Carbon isotope end-members for mixing plot curves shown in Fig. 6

**Water mass**	**$\delta^{13}C$ (‰)**	**Reference**	**[DIC] (μmol kg^{-1})**	**Reference**
NADW	1.3	[40]	2180	[41]
AABW	0.4	[23]	2280	[41]
GNADW	1.5	[42]	2180	Assumed constant
GAABW	-0.5	[36,39,43]	2390	DIC to $\delta^{13}C$ [44]

Supplementary Note 1

To construct the cross plots of benthic foraminiferal $\delta^{13}C$ versus ε_{Nd} in Fig. 6 only cores from the Antarctic Bottom Water mixing zone in Supplementary Fig. 2 were included to limit the interpretation to binary mixing. $\delta^{13}C$ data and their sources are listed in Supplementary Table 7. The neodymium end-member compositions were taken from Supplementary Fig. 2 whilst the $\delta^{13}C$ end-member values and references are given in Supplementary Table 8. The $\delta^{13}C$ of North Atlantic Deep Water was taken from seawater data corrected for the Suess Effect[40]. The possibility of a glacial northern-sourced water mass with low $\delta^{13}C$ was discounted on the basis of a lack of evidence from benthic foraminiferal $\delta^{13}C$ values from the intermediate-depth North Atlantic and the deep Nordic Seas[45,46]. The estimated $\delta^{13}C$ of Glacial Antarctic Bottom Water (GAABW) was obtained by applying the same -0.4‰ offset between seawater and benthic foraminiferal $\delta^{13}C$ observed in the South Atlantic values in the Holocene cross plot to glacial benthic foraminiferal $\delta^{13}C$ values. This offset is attributed to the Mackensen Effect, whereby benthic foraminifera preserve $\delta^{13}C$ values lower than ambient seawater values due to the effect of a benthic nepheloid layer containing carbon with a $\delta^{13}C$ lower than that of the overlying seawater. This phenomenon is most common in regions where there is high surface productivity and therefore a large delivery of organic carbon to the sea floor[43]. Applying this correction for the Mackensen Effect gave a GAABW $\delta^{13}C$ of -0.5‰, in good agreement with Curry and Oppo[46] who predicted a $\delta^{13}C$ of at least -0.5‰ for GAABW, but stated that it could be as negative as -1‰. The dissolved inorganic carbon (DIC) concentration of GAABW was estimated using the relationship between seawater $\delta^{13}C$ and DIC (Supplementary Equation 1), where $\delta^{13}C_{org}$ = -20‰[44]. Mixing between the end-members in each plot was calculated using Equation 1 for neodymium, and the equivalent equation modified to include $\delta^{13}C$ and DIC instead of the neodymium parameters for carbon.

Supplementary Equation 1. $\delta^{13}C_{Modern}\,[DIC]_{Modern} + \delta^{13}C_{Org}\,[DIC]_{Org} = \delta^{13}C_{Glacial}\,[DIC]_{Glacial}$

Supplementary References:

1. Piepgras, D. J. & Wasserburg, G. J. Rare earth element transport in the western North Atlantic inferred from Nd isotopic observations. *Geochim. Cosmochim. Acta* **51,** 1257–1271 (1987).

2. Foster, G. L., Vance, D. & Prytulak, J. No change in the neodymium isotope composition of deep water exported from the North Atlantic on glacial-interglacial time scales. *Geology* **35,** 37–40 (2007).

3. Stichel, T., Frank, M., Rickli, J. & Haley, B. A. The hafnium and neodymium isotope composition of seawater in the Atlantic sector of the Southern Ocean. *Earth Planet. Sci. Lett.* **317-318,** 282–294 (2012).

4. Skinner, L. C. *et al.* North Atlantic versus Southern Ocean contributions to a deglacial surge in deep ocean ventilation. *Geology* **41,** 667–670 (2013).

5. Amakawa, H., Sasaki, K. & Ebihara, M. Nd isotopic composition in the central North Pacific. *Geochim. Cosmochim. Acta* **73,** 4705–4719 (2009).

6. Noble, T. L., Piotrowski, A. M. & Mccave, I. N. Neodymium isotopic composition of intermediate and deep waters in the glacial southwest Pacific. *Earth Planet. Sci. Lett.* **384,** 27–36 (2013).

7. Orsi, A. H., Whitworth, T. & Nowlin, W. D. On the meridional extent and fronts of the Antarctic Circumpolar Current. *Deep Sea Res. Part I Oceanogr. Res. Pap.* **42,** 641–673 (1995).

8. Marcott, S. A. *et al.* Centennial-scale changes in the global carbon cycle during the last deglaciation. *Nature* **514,** 616–619 (2014).

9. Roberts, N. L., Piotrowski, A. M., McManus, J. F. & Keigwin, L. D. Synchronous deglacial overturning and water mass source changes. *Science* **327,** 75–78 (2010).

10. McManus, J. F., Francois, R., Gherardi, J.-M., Keigwin, L. D. & Brown-Leger, S. Collapse and rapid resumption of Atlantic meridional circulation linked to deglacial climate changes. *Nature* **428,** 834–837 (2004).

11. Stuiver, M. & Grootes, P. M. GISP2 Oxygen Isotope Ratios. *Quat. Res.* **53,** 277–284 (2000).

12. Curry, W. B. & Lohmann, G. P. Carbon isotopic changes in benthic foraminifera from the western South Atlantic: Reconstruction of glacial abyssal circulation patterns. *Quat. Res.* **18,** 218–235 (1982).

13. Lippold, J. *et al.* Strength and geometry of the glacial Atlantic Meridional Overturning Circulation. *Nat. Geosci.* **5,** 813–816 (2012).

14. Hendry, K. R. *et al.* Abrupt changes in high-latitude nutrient supply to the Atlantic during the last glacial cycle. *Geology* **40,** 123–126 (2012).

15. Chen, M.-T. *et al.* Late Quaternary sea-surface temperature variations in the southeast Atlantic: a planktic foraminifer faunal record of the past 600 000 yr (IMAGES II MD962085). *Mar. Geol.* **180,** 163–181 (2002).

16. Pichevin, L., Cremer, M., Giraudeau, J. & Bertrand, P. A 190 ky record of lithogenic grain-size on the Namibian slope: Forging a tight link between past wind-strength and coastal upwelling dynamics. *Mar. Geol.* **218,** 81–96 (2005).

17. Daniau, A.-L. *et al.* Orbital-scale climate forcing of grassland burning in southern Africa. *Proc. Natl. Acad. Sci.* **110,** 5069–5073 (2013).

18. Bird, M. I. & Cali, J. A. A revised high-resolution oxygen-isotope chronology for ODP-668B: Implications for Quaternary biomass burning in Africa. *Glob. Planet. Change* **33,** 73–76 (2002).

19. Noble, T. L. Southern Ocean circulation and sediment sourcing. (University of Cambridge, 2012).

20. Prell, W. L. *et al.* Surface circulation of the Indian Ocean during the last glacial maximum, approximately 18,000 yr B.P. *Quat. Res.* **14,** 309–336 (1980).

21. CLIMAP Project Members. Seasonal reconstruction of the Earth's surface at the last glacial maximum. *Geol. Soc. Am. Map Chart Ser.* 1–18 (1981).

22. Curry, W. B., Duplessy, J., Labeyrie, L. & Shackleton, N. J. Changes in the distribution of $\delta^{13}C$ of deep water CO_2 between the last glaciation and the Holocene. *Paleoceanography* **3,** 317–341 (1988).

23. Oppo, D. W. & Fairbanks, R. G. Variability in the deep and intermediate water circulation of the Atlantic Ocean during the past 25,000 years: Northern Hemisphere modulation of the Southern Ocean. *Earth Planet. Sci. Lett.* **86,** 1–15 (1987).

24. Chapman, M. R. & Shackleton, N. J. Millennial-scale fluctuations in North Atlantic heat flux during the last 150,000 years. *Earth Planet. Sci. Lett.* **159,** 57–70 (1998).

25. Hodell, D. A., Venz, K. A., Charles, C. D. & Ninnemann, U. S. Pleistocene vertical carbon isotope and carbonate gradients in the South Atlantic sector of the Southern Ocean. *Geochemistry, Geophys. Geosystems* **4,** 1–19 (2003).

26. Shackleton, N. J. *et al.* The Oxygen Isotope Stratigraphic Record of the Late Pleistocene [and Discussion]. *Philos. Trans. R. Soc. B Biol. Sci.* **280,** 169–182 (1977).

27. Balsam, L. & McCoy, F. W. Atlantic Sediments: Glacial/Interglacial Comparisons. *Paleoceanography* **2,** 531–542 (1987).

28. Bé, A. W. H., Damuth, J. E., Lott, L. & Free, R. Late Quaternary Climatic Record in Western Equatorial Atlantic Sediment. *Geol. Soc. Am. Mem.* **145,** 165–200 (1976).

29. Mix, A. C. & Fairbanks, R. G. North Atlantic surface-ocean control of Pleistocene deep-ocean circulation. *Earth Planet. Sci. Lett.* **73,** 231–243 (1985).

30. Wei, R., Abouchami, W., Zahn, R. & Masque, P. Deep circulation changes in the South Atlantic since the Last Glacial Maximum from Nd isotope and multi-proxy records. *Earth Planet. Sci. Lett.* **434,** 18–29 (2016).

31. Jonkers, L. *et al.* Deep circulation changes in the central South Atlantic during the past 145 kyrs reflected in a combined ^{231}Pa/230Th, Neodymium isotope and benthic δ^{13}C records. *Earth Planet. Sci. Lett.* **419,** 14–21 (2015).

32. Gutjahr, M., Frank, M., Stirling, C. H., Keigwin, L. D. & Halliday, A. N. Tracing the Nd isotope evolution of North Atlantic Deep and Intermediate Waters in the western North Atlantic since the Last Glacial Maximum from Blake Ridge sediments. *Earth Planet. Sci. Lett.* **266,** 61–77 (2008).

33. Huang, K.-F., Oppo, D. W. & Curry, W. B. Decreased influence of Antarctic intermediate water in the tropical Atlantic during North Atlantic cold events. *Earth Planet. Sci. Lett.* **389,** 200–208 (2014).

34. Piotrowski, A. M. *et al.* Reconstructing deglacial North and South Atlantic deep water sourcing using foraminiferal Nd isotopes. *Earth Planet. Sci. Lett.* **357-358,** 289–297 (2012).

35. Lang, D. C. *et al.* Incursions of southern-sourced water into the deep North Atlantic during late Pliocene glacial intensification. *Nat. Geosci.* In press (2016). doi:10.1038/ngeo2688

36. Waelbroeck, C. *et al.* The timing of deglacial circulation changes in the Atlantic. *Paleoceanography* **26,** PA3213 (2011).

37. Boyle, E. A. & Keigwin, L. D. North Atlantic thermohaline circulation during the past 20,000 years linked to high-latitude surface temperature. *Nature* **330,** 35–40 (1987).

38. Boyle, E. A. Cadmium and δ^{13}C paleochemical ocean distributions during the Stage 2 Glacial Maximum. *Annu. Rev. Earth Planet. Sci.* 245–287 (1992).

39. Ninnemann, U. S. & Charles, C. D. Changes in the mode of Southern Ocean circulation over the last glacial cycle revealed by foraminiferal stable isotopic variability. *Earth Planet. Sci. Lett.* **201,** 383–396 (2002).

40. Olsen, A. & Ninnemann, U. Large $\delta^{13}C$ gradients in the Preindustrial North Atlantic Revealed. *Science* **330,** 658–659 (2010).

41. Broecker, W. S. & Peng, T.-H. The Cause of the Glacial to Interglacial Atmopheric CO_2 Change: A Polar Alkalinity Hypothesis. *Global Biogeochem. Cycles* **3,** 215–239 (1989).

42. Slowey, N. C. & Curry, W. B. Glacial-interglacial differences in circulation and carbon cycling within the upper western North Atlantic. *Paleoceanography* **10,** 715–732 (1995).

43. Mackensen, A., Hubberten, H., Bickert, T. & Ftitterer, D. K. The $\delta^{13}C$ in benthic foraminifera tests of Fontbotia wuellerstorfi (Schwager) relative to the $\delta^{13}C$ of dissolved inorganic carbon in Southern Ocean deep water: implications for glacial ocean circulation models. *Paleoceanography* **8,** 587–610 (1993).

44. Kroopnick, P. The distribution of ^{13}C of ΣCO_2 in the world oceans. *Deep. Res.* **32,** 57–84 (1985).

45. Veum, T., Jansen, E., Arnold, M., Beyer, I. & Duplessy, J.-C. Water mass exchange between the North Atlantic and the Norwegian Sea during the past 28,000 years. *Nature* **356,** 783–785 (1992).

46. Curry, W. B. & Oppo, D. W. Glacial water mass geometry and the distribution of $\delta^{13}C$ of ΣCO_2 in the western Atlantic Ocean. *Paleoceanography* **20,** PA1017 (2005).
